# Perinatal Light Imprinting of Circadian Clocks and Systems (PLICCS): The PLICCS and Cancer Hypothesis

**DOI:** 10.3389/fonc.2017.00044

**Published:** 2017-03-20

**Authors:** Philip Lewis, Thomas C. Erren

**Affiliations:** ^1^Institute and Policlinic for Occupational Medicine, Environmental Medicine and Prevention Research, University Hospital of Cologne, Cologne, Germany

**Keywords:** circadian, perinatal, photoperiod, chronodisruption, season, latitude, epidemiology, cancer prevention

## Abstract

Circadian disruption is associated with sleep, mood, and metabolic disorders, and—according to the International Agency for Research on Cancer—even with cancer. Mechanistically, the source of disease may be circadian system instability which likely arises during development. In animal experiments, both low perinatal light:dark ratios and chronic perinatal photoperiod phase shifting yield enduring, detrimental effects on neuroendocrine physiology *via* circadian system instability. Certainly, accumulating disturbances to neuroendocrine physiology and detrimental downstream effects could predispose to internal cancers. Epidemiologically, either season of birth or latitude of birth, both of which co-determine perinatal photoperiod-zeitgeber strengths, have been utilized independently as proxies for other environmental co-etiologies of cancer. Both have been independently associated with cancer; however, the evidence is inconclusive. We hypothesize that time of birth and location of birth, together determining perinatal photoperiod, contribute to cancer development through *P*erinatal *L*ight *I*mprinting of *C*ircadian *C*locks and *S*ystems.

## Background

Plants and animals match their physiology and behavior to the 24-h solar photoperiod. Neuroendocrine rhythmicity, the internal form of communication about—and response to—external time, must constantly re-align to photoperiods which change over days and seasons. When confined to rather constant geographical locations, this constant re-alignment is very gradual and consciously imperceptible. An abrupt circadian disruption such as a trans-meridian flight, whereby the endogenously anticipated photoperiod dramatically changes, has perceptible effects on physiology which we know as jet-lag, and it can take several days for our internal (biological) time to re-align with external (environmental) time. Circadian disruption can affect sleep, mood, and the temporal organization of physiology. Accumulated circadian disruption to physiology is associated with sleep, mood, metabolic disorders, and even cancer (1). As to use of the term cancer generically, there are two reasons why we should not restrict to specific types of cancer in the present manuscript: (1) circadian clocks and systems pervade almost every cell in the human body governing growth and metabolic processes, and (2) many endocrine signaling factors with different organ and tissue targets are under circadian control.

## Experimental Links Between Perinatal Photoperiods and the Development of Circadian Clocks and Systems

The development of circadian clocks and systems is susceptible to environmental factors, particularly light (2). In mice, different perinatal photoperiod exposures have been shown to effect differential neuronal firing parameters and circadian rhythms of gene expression of the suprachiasmatic nuclei (the internal master circadian clock) (3), circadian activity behavior, and the ability to anticipate dusk—effects that persist regardless of the continuation photoperiod (4, 5). Perinatal light affects development of mouse and rat circadian motor activities and adult rat behavior (6–8). Growth and behavior in the Siberian hamster is affected by the interaction between the perinatal and continuation photoperiods in a sex-dependent manner (9). Recent studies have shown that perinatal light environments affect how adult mouse and rat circadian behaviour is shaped by different photoperiods and, further, that perinatal photoperiod challenges that abruptly disrupt and phase shift endogenous circadian rhythms cause neuroendocrine and metabolic derangement in adulthood in Wistar rats. These changes are observed despite being maintained on controlled lighting schedules for the next 12 months and some are sex dependent (2, 10–14). Many endocrine signaling factors that are under circadian control and have a prominent role in transmitting circadian information are key to growth and development processes. Hormone and metabolic imbalance can be linked to various types of internal cancers and thus, by extension, so can circadian instability. Additionally, circadian instability in expression of genes and proteins governing cell growth, metabolism, and homeostasis could also predispose to cancer. In agreement with the concepts of “environmental imprinting of the mammalian circadian clock and its response to subsequent seasonal change under seasonal light cycles” by Ciarleglio et al. (4) and “increased vulnerability to circadian disruption” by Ohta et al. (5), *P*erinatal *L*ight *I*mprinting of *C*ircadian *C*locks and *S*ystems (PLICCS) results in higher or lower susceptibilities to exogenous and endogenous circadian challenges later in life which may result in sex-dependent differences in neuroendocrine, metabolic, and growth disruption, and therefore potentially different types of internal cancer.

## Epidemiological Links Between Season or Latitude of Birth and Cancer

Season of birth or latitude of birth which together determine perinatal photoperiods and zeitgeber strengths [zeitgebers are external factors that synchronize an individual’s biological rhythms with the environment; first defined by Aschoff (15, 16); in this case, referring to light:dark ratio and light intensities entraining circadian clocks] have been studied each on its own with regard to different types of cancer but have not been studied together.

In effect, evidence for either being associated with cancer development is conflicting, the relevance of photoperiod is not sufficiently discussed, and authors have attributed findings to other environmental factors (17–21). Candidates to explain conflicting findings [for example, evidence of seasonal clustering of births in cancer patients as opposed to no evidence of seasonal clustering (18, 19, 21)] likely relate to differences in studying combined neoplasms as opposed to differentiating specific cancers, genetic susceptibility (or lack thereof) within small regions or distinct populations, and heterogeneous challenges presented to circadian clocks and systems. Importantly, there is evidence which is compatible with the PLICCS rationale, such as a winter birth clustering for acute myeloid leukemia in Sweden or a latitudinal gradient for lymphoma subtypes in Australia (18, 20). Sex differences have also been observed.

## Hypothesis

We hypothesize that—after taking sex, specific cancer types, and post-perinatal challenges into account—time and location of birth contribute to—and may predict—cancer development in later life through PLICCS.

**Figure 1:**
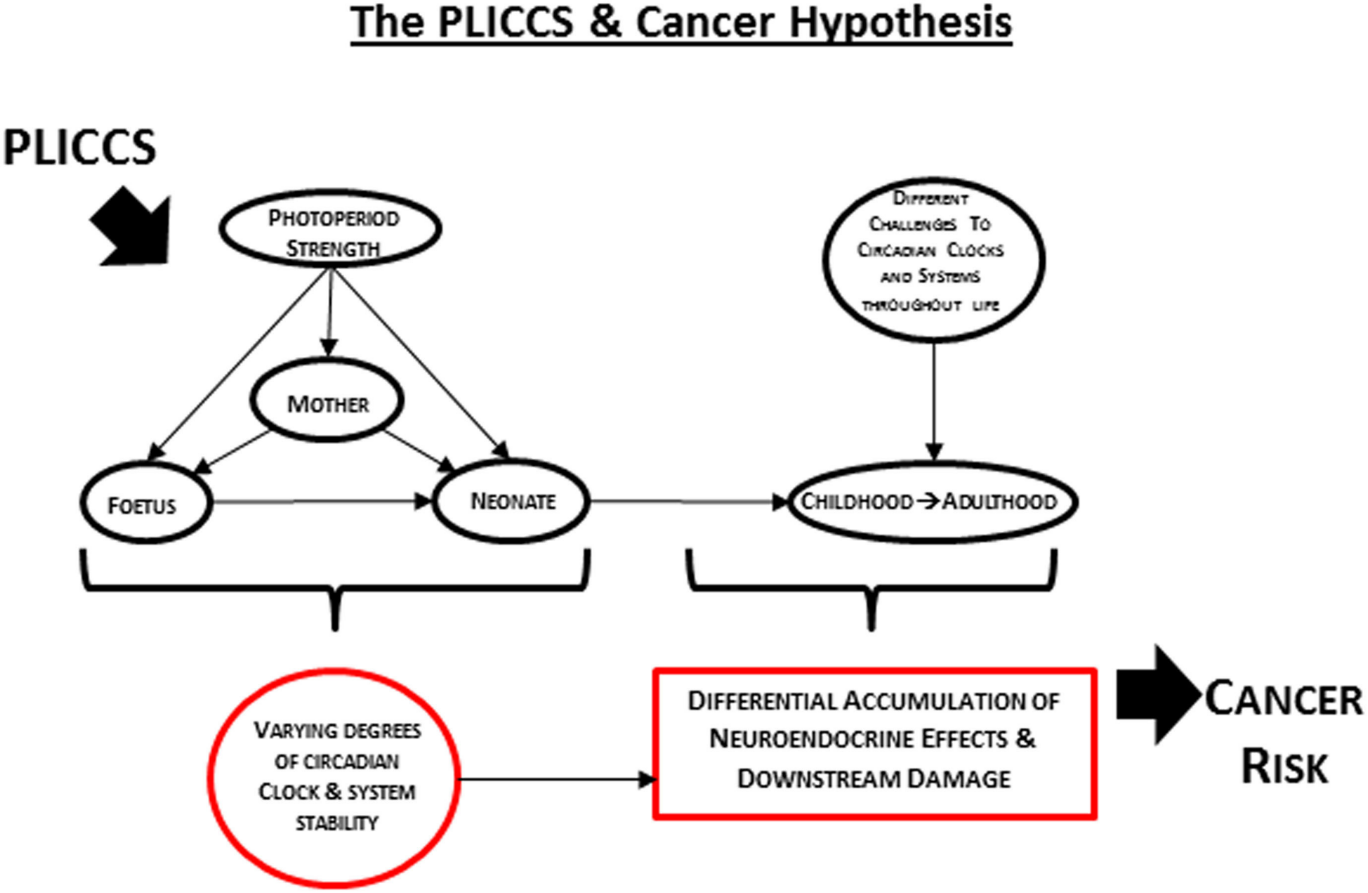
**Illustration of the PLICCS and cancer hypothesis**.

In 2012, it was postulated that humans born and raised postnatally (and we propose here that PLICCS incorporates 3 months prior to and after birth) under conditions of low zeitgeber strength are at greater risk of developing internal cancer than those born and raised under conditions of high zeitgeber strength (22). Based on the supporting evidence described above, we build here on this previous postulate to propose the PLICCS and cancer hypothesis: sex-specific associations of different cancer types with different perinatal zeitgeber strength exposures will be increasingly positive with greater degrees of post-perinatal challenges and accumulation of neuroendocrine effects and downstream damage mediated through disruptions of circadian clocks and systems stability (Figure 1).

## Testing the Hypothesis

For epidemiological testing of this hypothesis, three portions of information are necessary: (a) The perinatal zeitgeber strength; (b) (Co-)determinants of cancer—both established and suspected; (c) Ancillary information (covariates, including potential confounders and effect modifiers).

In regard to (a)–(c), it is clear that data concerning season of birth and latitude of birth, as well as cancer type, progression, age, and sex of patient must be acquired. Equally clearly, relevant confounding data such as exposure to other known cancer risk factors (e.g., smoking, family history of cancer, alcohol, infection, exposure to cancerous agents, and chemicals) need to be taken into account.

In specific regard to (a), different zeitgeber strengths according to season and latitude at and around birth must be adequately defined. For example, how does 16 h of lower intensity sunlight closer to the Arctic compare to 10 h of higher intensity sunlight closer to the tropics? Ideally, average lux for a certain perinatal period, such as 3 months before and after birth, and place would be determined from meteorological data. Were this data not available, other suggestions to overcome the relative zeitgeber strength problem might be to use three-dimensional models for analysis (*x, y*, and *z* for latitude of birth, season of birth, and cancer variable, respectively). Based on the empirical information on possible links between perinatal zeitgeber strength and an imprinting of circadian system stability, L:D ratios examined by Ciarleglio et al. and Ohto et al. (4, 5), i.e., 8:16, 12:12, 16:8, and24:0, would take on the value range 0.5 ≤ 1 ≤ 2 < 24. While it may take many years to understand the details, L:D 12:12 may be a reference point in humans as has been in the study by Ciarleglio et al. for mice. This would render low and high zeitgeber strengths to correspond with L:D ratios below or above 1 and close to 0.5 and 2, respectively.

In specific regard to (b) and (c), beyond other challenges, such as shift-work, to circadian clocks and systems which are more or less stable, experimental evidence suggests that the post-perinatal photoperiod challenge should be considered where possible in relation to the PLICCS cancer hypothesis (4). A less stable circadian system resultant from PLICCS may cope with gradual seasonal changes of photoperiods and less extremes between seasons equally well as a more stable circadian system with more extreme differences between seasons. With regard to cancer, pineal melatonin is secreted in greater amounts in humans living at higher latitudes in winter due to the prolonged dark period and may play a role in shaping correlations of season and latitude of birth with cancer occurrence due to suggested potential oncostatic properties (17). It may be that different PLICCS and cancer relationships are observed within ranges of latitudes rather than across entire hemispheres.

Importantly, if there are increased risks for specific types of PLICCS-induced cancers rather than internal cancers in general, there are no data available to help us reliably differentiate between all internal cancer types. Indeed, it is only with studies such as PLICCS and internal cancer generally that we may identify specific PLICCS-causing types of cancer.

One relevant question is “could photic exposures beyond natural light alter the perinatal zeitgeber strength”? We expect that natural light with intensities of 20,000 to 100,000 lux from winter through summer determines zeitgeber strength; insofar we expect to identify PLICCS effects—if they exist—despite man-made light ranging from few lux in residential settings to 500 lux as a standard requirement in many occupational settings. Empirically, a recent study by Bauer and colleagues, while being focused on a psychiatric endpoint, has been the first epidemiological study to conclusively report data targeted at the PLICCS rationale (23). In principle, cohort studies can be used to systematically examine whether psychiatric disease rates in individuals born in winter months, adjusted for latitudinal photoperiod, are higher than in individuals born at other times of the year (22, 24, 25); case–control studies can explore whether the likelihood of having been born in winter months is higher in cases with psychiatric diseases than in controls without the disease. That this type of study can be performed for a psychiatric endpoint means similar pooled cohort data can be employed for a cancer endpoint. With specific regard to cancer endpoints, we are currently investigating options to test predictions based on the PLICCS hypothesis within large international cohort collaborations. International cohort collaborations such as the I4C (The International Childhood Cancer Cohort Consortium) (26) offer large-scale prospective information which may be used for (a)–(c) above; note that enrollment of people at birth for one cohort began as early as 1964. The I4C is a highly reputable consortium of cohort representatives who published hundreds of epidemiological studies into possible consequences of early environmental and other exposures for study individuals’ cancer or other diseases in later life stages. These databases can allow exploring whether certain variables, both environmental and genetic, can explain and possibly predict differential onsets and courses of cancer in humans who are followed from their perinatal period.

Moreover, researchers who studied relationships of either season or latitude with cancer occurrence could re-visit completed studies as a time- and cost-efficient research option or launch novel studies, which employ the time and location of birth as a variable to explain, and possibly predict, differential onsets and courses of disease. Provided that epidemiological studies were to yield results compatible with the PLICCS hypothesis and associated predictions, note that studies in individuals with perinatal light exposures at or around the equator could serve as control populations; indeed, homogeneous exposures to L:D 12:12 at the equator would imply that PLICCS metrics should not explain or predict differential cancer occurrence later in life which we hypothesize for individuals who experience gradients of perinatal light-associated zeitgeber strength.

## Conclusion—What if the Hypothesis is Valid?

Let us assume that PLICCS, depending on low vs. high zeitgeber strength, can be either a cancer risk or protective factor. In that case, we could arrange for perinatal summer light exposure conditions irrespective of time of year and geographical location. Indeed, light represents a very amenable environmental agent for preventative measures against noxious-photic stimuli (27). Pregnant and breast-feeding mothers could utilize anthropogenic light to mimic a summer photoperiod during winter for themselves and their children. Furthermore, it has already been proposed to provide cyclical lighting for newborn intensive care units (28, 29). These lighting conditions could potentially be modified to reduce the risk of developing cancer later in life. Further studies would be required to determine optimum perinatal light conditions for preventative measures.

Associations between circadian disruption and cancer and between light and cancer have been reported ~half a century ago (30, 31). Somewhat scattered data from animal and human studies followed which is compatible with the PLICCS and cancer hypothesis such that the hypothesis warrants systematic testing. Should the PLICCS and cancer hypothesis hold true, simple preventative steps against cancer development would be at minimal cost while contributing to decrease what is a very large, and growing, global health system strain and economic burden.

## Author Contribution

PL and TE contributed equally to this manuscript.

## Conflict of Interest Statement

The authors declare that the research was conducted in the absence of any commercial or financial relationships that could be construed as a potential conflict of interest.
